# The change of healthcare service in Chinese patients with inflammatory bowel disease during the pandemic: a national multicenter cross-sectional study

**DOI:** 10.1038/s41598-023-46892-5

**Published:** 2023-11-16

**Authors:** Xiaofei Li, Fang Wang, Yizhen Jia, He Zhou, Yanting Shi, Feng Tian, Yan Chen, Jie Liang

**Affiliations:** 1 State Key Laboratory of Holistic Integrative Management of Gastrointestinal Cancers and National Clinical Research Center for Digestive Diseases, Xijing Hospital of Digestive Diseases, Fourth Military Medical University, Changle West Road No.169, Xi’an, 710032 Shaanxi China; 2 School of Public Health, Capital Medical University, Anzhen Road No.2, Chaoyang District, Beijing, 100069 China; 3 Department of Gastroenterology, Shengjing Hospital of China Medical University, Sanhao Street No.36, Shenyang, 110004 Liaoning China; 4 Department of Gastroenterology, The Second Affiliated Hospital, School of Medicine, Zhejiang University, Jiefang Road No.88, Hangzhou, 310009 Zhejiang China

**Keywords:** Inflammatory bowel disease, Public health

## Abstract

The pandemic of COVID-19 was a major public health events and had a deeply impact on the healthcare acquired by patients with inflammatory bowel disease (IBD). The purpose of this study was to evaluate the long-term impacts on healthcare service in Chinese IBD patients under the dynamic zero-COVID strategy. The study was performed in the Inflammatory Bowel Disease Quality of Care Centers in mainland China in 2021. The data about the healthcare was collected by a 44-item questionnaire. Totally 463 were from ulcerative colitis (UC) patients and 538 from Crohn’s disease (CD) patients were included in the study. The pandemic impacted 37.5% patients on their treatment, and the biggest problem was unable to follow up timely (77.9%). There was a significant increase in healthcare costs in CD (*P* < 0.001) and no significant change in UC (*P* = 0.14) after the outbreak. Both UC and CD had an increase in the frequency of outpatient visits (UC 5.07 vs. 4.54, *P* = 0.001; CD 6.30 vs. 5.76, *P* = 0.002), and hospitalizations (UC 1.30 vs. 1.02, *P* < 0.001; CD 3.55 vs. 2.78, *P* < 0.001). The hospitalization rate in UC reduced slightly (40.2% vs. 42.8%, *P* = 0.423) after the outbreak, but it significantly increased in CD (75.8% vs. 67.8%, *P* = 0.004). The rate of biologics had significant increased (UC 11.2% vs. 17.7%, *P* = 0.005; CD 53.2% vs. 71.0%, *P* < 0.001). Besides, the proportion of people using telemedicine also increased from 41.6% to 55.1% (*P* < 0.001). However, 82.8% patients still preferred face-to-face visits. Recurrent outbreaks and the regular pandemic prevention and control policy had a long-term impact on medical care service for IBD patients. The preferred mode of healthcare was still face-to-face visit. It will be a long way to go in the construction of telemedicine in China.

## Introduction

The coronavirus disease 2019 (COVID-19) pandemic caused by the severe acute respiratory syndrome coronavirus 2 (SARS-CoV-2) has been ongoing for more than 3 years since December 2019. It has had a tremendous impact on the entire human community in all aspects. Although the advent of the COVID-19 vaccine has reduced the rate of severe disease and mortality from COVID-19, the rapid mutation of the virus and the recurrences of outbreaks continue to keep us anxious. Regular practices to prevent and control the outbreak of the pandemic have been applied in many countries. China’s dynamic zero-COVID strategy had been in place for almost 3 years. At the end of 2022, the Chinese government had modified the regulatory measures in the light of the emerging situation and rescinded the travel restrictions. Chinese society has entered the post-pandemic era.

Inflammatory bowel disease (IBD) is a chronic relapsing–remitting incurable gastrointestinal disease that requires long-term regular therapy and disease monitoring. Patients with IBD have a tripled risk of severe viral infections (ie. cytomegalovirus, Epstein-Barr virus, varicella zoster virus and herpes simplex virus)^1^. Previous study showed higher tissue concentrations of angiotensin-converting enzyme 2, a molecule that allowed SARS-CoV-2 stinger protein to bind host cells and infect humans, in patients with active ulcerative colitis (UC) and Crohn’s disease (CD)^2^. Meanwhile, immunosuppressive drugs are commonly used in the treatment of IBD, so IBD patients are considered to be a susceptible population for the SARS-CoV-2. Travelling restrictions were also another factor that could not be ignored. These adverse factors would undoubtedly affect treatment decisions for IBD patients during the COVID-19 pandemic. It was unknown how the medical care services received by IBD patients had changed in long-term in the real world after the outbreak in China. So we conducted a nationwide, multicenter cross-sectional study to study changes in the healthcare services for patients with IBD in China.

## Methods

### Study design

This study was a multiple centers cross-sectional observational study. The data was collected by a electronic questionnaire. According to the research purpose, the researchers who were experienced IBD physicians designed the questionnaire, which was revised by senior IBD specialists. A pre-survey was conducted and the details of the questionnaire were adjusted to make it easier for patients to answer.

The questionnaire content mainly includes basic demographic characteristics, disease-related information, treatment-related information, and telemedical service-related information in 2019 and 2020 (Supplemental appendix A). The electronic questionnaire was produced by Wenjuanxing (https://www.wjx.cn), which is a free and open platform for survey design. The survey was performed from April to December, 2021.

### Patient population

The inclusion criteria were: (i) age ≥ 18 years; (ii) diagnosed with IBD before 2019 according to the Chinese consensus on diagnosis and treatment of inflammatory bowel disease (Beijing, 2018); (iii) treated and followed up for more than 1 year at the IBD regional treatment centers and its regional alliance hospitals; (iv) able to understand and complete the electronic questionnaire independently.

Exclusion criteria were: (i) combined malignancy or other serious systemic diseases; (ii) in pregnancy or lactation; (iii) patients who participated in other clinical trials in the last 3 months.

### Definitions

Because the outbreak of the pandemic in China was in January 2020, we define 2019 as the pre-pandemic and 2020 as the post-pandemic period. Based on the domestic pandemic control in China, we further define the first half of 2020 as the period of outbreak and the second half of 2020 as the period of normalized prevention and control.

Telemedicine is defined as an online medical service platform provided by hospitals or third parties, which can offer online consultations, provide medical advice on diagnosis and treatment, and even electronically prescribe medications. Medical consultations with a physician that are conducted solely by telephone or other web-based chat tools are not considered telemedicine.

### Statistical analysis

In statistical analysis, continuous variables were presented as mean ± standard deviation or median (25th–75th percentiles) and categorical variables were presented as frequencies (percentages). The t-test or nonparametric test was used to compare continuous variables, and the chi-square test or Fisher’s exact test was used to compare categorical variables. The data were analyzed by SPSS 25.0 software (IBM). *P* values less than 0.05 were considered to be statistically significant.

### Ethics approval and consent to participate

This study was conducted according to the guidelines of the Declaration of Helsinki, and proved by the Chinese Ethics Committee of Registering Clinical Trials (reference number: ChiECRCT20210023). Informed consent was obtained from all patients in the first page of the questionnaire.

## Result

A total of 1064 questionnaires were collected, 63 of which were unqualified and excluded, and 1001 questionnaires were included in the study. Patients participating in the study were from all provinces in mainland China, except Tibet, Hainan province, Hong Kong and Macau.

### Demographic characteristics and disease related information of IBD patients

Demographic characteristics and disease related information of patients were reported in Table 1. CD patients showed a higher proportion of male (*P* = 0.004), younger (*P* < 0.001), single (*P* < 0.001), and higher educational level (*P* = 0.024), compared to UC patients. Seventy percent of IBD patients were more concerned about poor disease control than the 30% who were worried about being infected with SARS-CoV-2 during the pandemic. Meanwhile, UC patients are more worried about infection of SARS-CoV-2 than CD patients (34.8% vs. 25.3%, *P* = 0.01). Besides, there were only35.5% (355/1001) patients who were vaccinated against COVID-19 by the end of the questionnaire. No patients were reported infection with covid-19 during the time of our survey.

**Table 1:** Demographics and disease related information of patients.

	Total N = 1001	UC n = 463	CD n = 538	P*
Mean age, years ± SD	38.76 ± 12.4	43.62 ± 11.96	34.59 ± 11.20	< 0.001^a^
Sex [male], n [%]	610 [60.9%]	260 [56.2%]	350 [65.1%]	0.004^b^
Single, n [%]	288 [28.8%]	61 [13.2%]	227 [38.5%]	< 0.001^b^
Educational level, n [%]				
High school and below	444 [44.4%]	223 [48.2%]	221 [41.1%]	0.024^b^
College and above	557 [55.6%]	240 [51.8%]	317 [58.9%]	
Disease duration, n [%]				
< 5 yaers	579 [57.8%]	273 [59.0%]	306 [56.5%]	0.208^b^
5 to < 10 years	252 [25.2%]	106 [22.9%]	146 [27.1%]	
10 to < 15 years	92 [9.2%]	41 [8.8%]	51 [9.6%]	
≥ 15 years	78 [7.8%]	43 [9.3%]	35 [6.5%]	
Vaccinate against COVID-19	355 [35.5%]	177 [38.2%]	178 [33.1%]	0.090^b^
Spotlight after the outbreak, n [%]				
Poorly controlled disease	704 [70.3%]	302 [65.2%]	402 [74.7%]	0.001^b^
SARS-CoV-2 infection	297 [29.7%]	161 [34.8%]	136 [25.3%]	
Positive attitude to life, n [%]	833 [83.2%]	391 [84.4%]	442 [82.2%]	0.333^b^
Preferred mode of treatment, n [%]				
MWM	459 [45.9%]	176 [38.0%]	283 [52.6%]	< 0.001^b^
TCM	32 [3.2%]	12 [2.6%]	20 [3.7%]	
Combination of MWM and TCM				
Concerns during treatment, n [%]	510 [50.9%]	275 [59.4%]	235 [43.7%]	
Poorly controlled disease	502 [50.1%]	236 [51.0%]	266 [49.4%]	0.609^b^
Expensive treatment costs	372 [37.2%]	165 [35.6%]	207 [38.5%]	
Drug side effects	127 [12.7%]	62 [13.4%]	65 [12.1%]	
Stable disease after oubreak, n [%]	669 [66.8%]	292 [63.1%]	377 [70.1%]	0.019^b^
If therapy affected after the break, n [%]	375 [37.5%]	169 [36.5%]	206 [38.3%]	0.560^b^

^a^Calculated using Student t test.

^b^Calculated using chi-square test.

MWM: Modern western medicine; TCM: Traditional Chinese Medicine.

In terms of treatment mode, 45.8% (459/1001) patients preferred modern western medicine (MWM), 3.2% (32/1001) traditional Chinese medicine (TCM), and 50.9% (510/1001) combination of MWM and TCM. The proportion of combination of MWM and TCM was significantly higher in UC than that in CD (59.4% vs. 43.7%, *P* < 0.001), and the proportion of MWM was higher in CD than that in UC (52.6% vs. 38.0%, *P* < 0.001). In the course of treatment, 50.1% (502/1001) were concerned about disease state, 37.2% (372/1001) about expensive cost, and 12.7% (127/1001) about drug side effects. There was no significant difference between UC and CD in this regard (*P* = 0.609).

### Healthcare information in IBD patients

**Figure 1:**
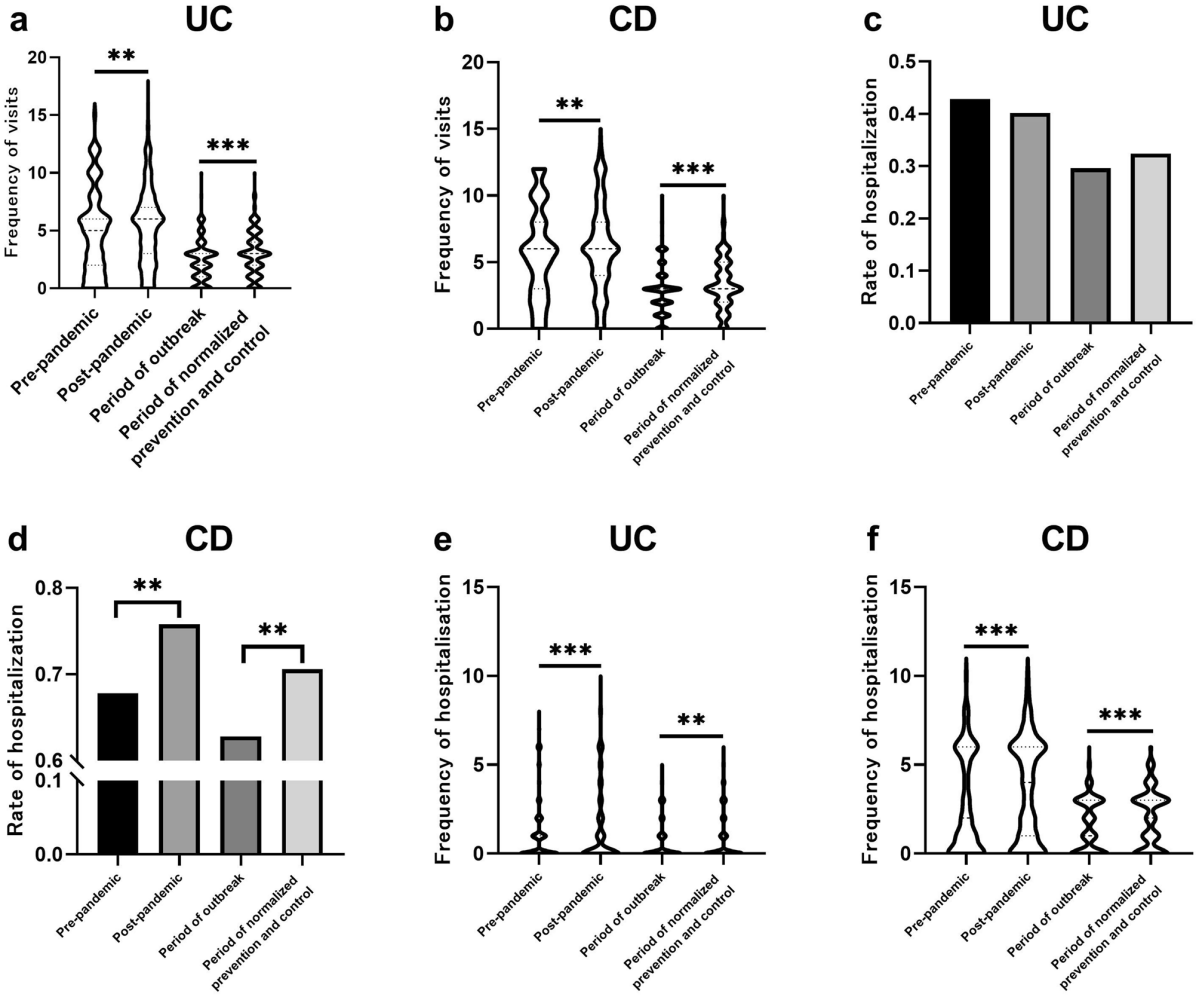
The frequency of visits, the rate of hospitalization and the frequency of hospitalization in patients with UC and CD. (**a**) the frequency of visits in UC; (**b**) the frequency of visits in CD; (**c**) the rate of hospitalization in UC; (**d**) the rate of hospitalization in CD; e: the frequency of hospitalization in UC; f: the frequency of hospitalisation in CD.

The frequency of visits shows a significant increase in patients with UC and CD (Fig. 1a, b). The median and mean frequency of visits in UC were 4 (2, 6) and 4.54 times/person-year before the outbreak, and 5 (2, 7) and 5.07 times/person-year in the first year of the pandemic, with a statistically significant difference (*P* = 0.001). Similarly, the median and mean frequency of visits in CD were 6 (3, 8) and 5.76 times/person-year before the outbreak, and 6 (5, 8) and 6.30 times/person-year in the first year of the pandemic, with a statistically significant difference (*P* = 0.002). There was also a significant increase in the frequency of visits in the period of normalized prevention and control compared to it in the period of the outbreak (UC, 2.27 vs. 2.80, *P* < 0.001; CD, 2.84 vs. 3.45, *P* < 0.001).

As regards the rates of hospitalization, there was no significant increase before and after the pandemic, remaining at around 40% (Fig. 1c). In contrast, the rate of hospitalization in CD was increased significantly after the outbreak (Fig. 1d), from 67.8 to 75.8% (*P* = 0.004), and was significantly higher in the period of normalized prevention and control than in the period of the outbreak (70.6% vs. 62.8%, *P* = 0.007). The change in the frequency of hospitalization followed the same trend in frequency of visits (Fig. 1e, f).

The percentage of UC patients operated on before and after the outbreak was 0.4%(2/463) and 0.9%(4/463) (*P* = 0.686) and the percentage of CD operated on was 10.4%(56/538) and 9.5%(51/538) (*P* = 0.611).

**Figure 2:**
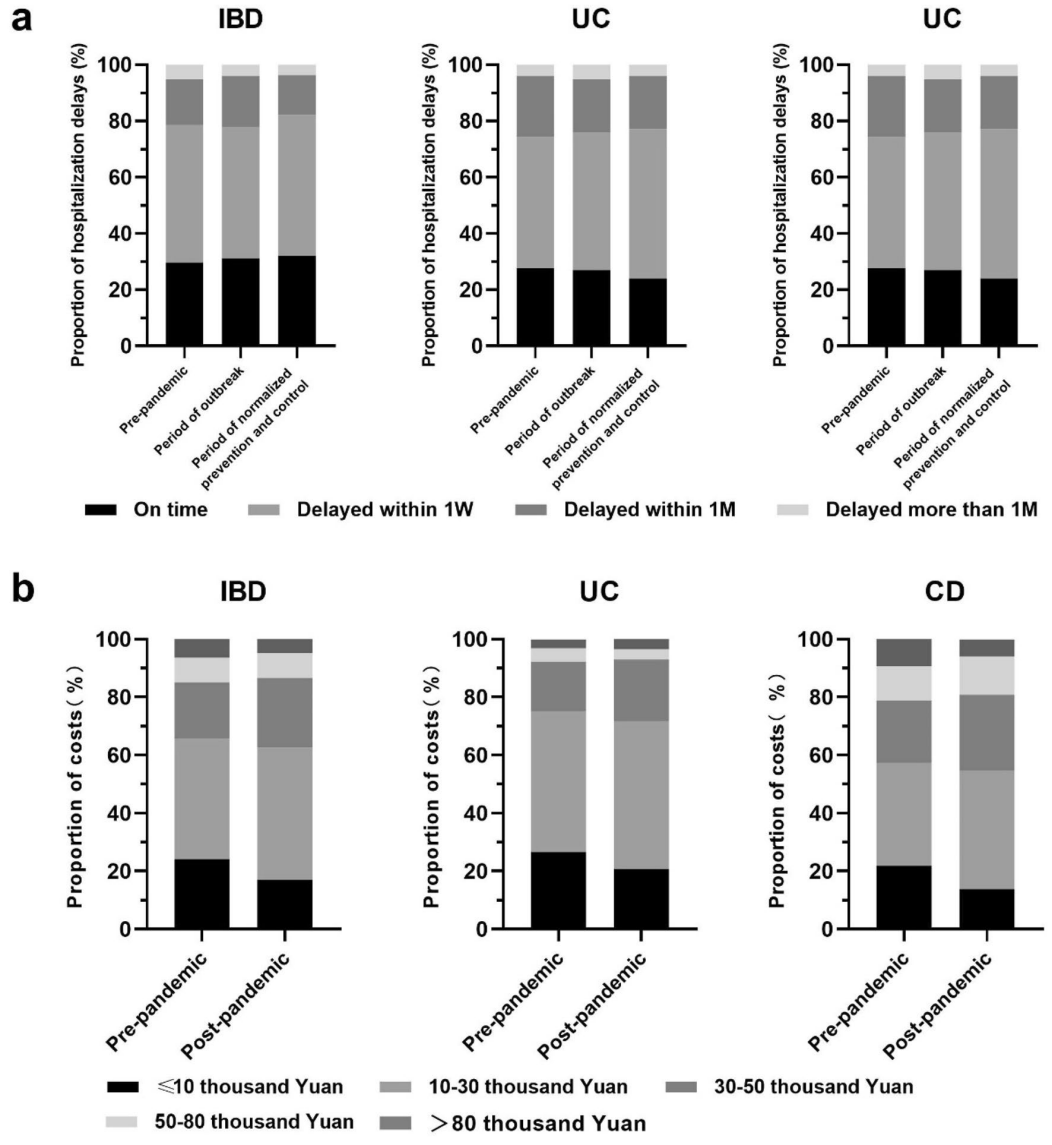
The healthcare change in the waiting time of hospitalization and disease burden in IBD patients. (**a**) Change in the waiting time of hospitalization; (**b**) Change in disease financial burden.

As we could see in Fig. 2a, the proportion of being hospitalized on time were 29.7%, 31.2%, and 32.1% in the pre-pandemic, outbreak, and the stage of the regular pandemic prevention and control, respectively. The majority of patients had a delayed admission within 1 week, accounting for 49.0%, 46.7%, and 50.2% respectively, but these differences were not statistically significant (*P* = 0.508).

The healthcare cost in CD patients increased significantly compared with that before the pandemic (χ^2^ = 25.99, *P* < 0.001) (Fig. 2b). The increase was mainly seen in the proportion of groups spending 10–30 thousand Yuan per year and 30–50 thousand Yuan per year, which were increased from 35.5% to 41.5% and 21.4% to 26.9% respectively. By contrast, the change of healthcare cost was not significant in UC patients (χ^2^ = 6.92, *P* = 0.14). Overall, the disease burden in the IBD patients increased compared to the pre-pandemic (χ^2^ = 25.67, *P* < 0.001).

**Figure 3:**
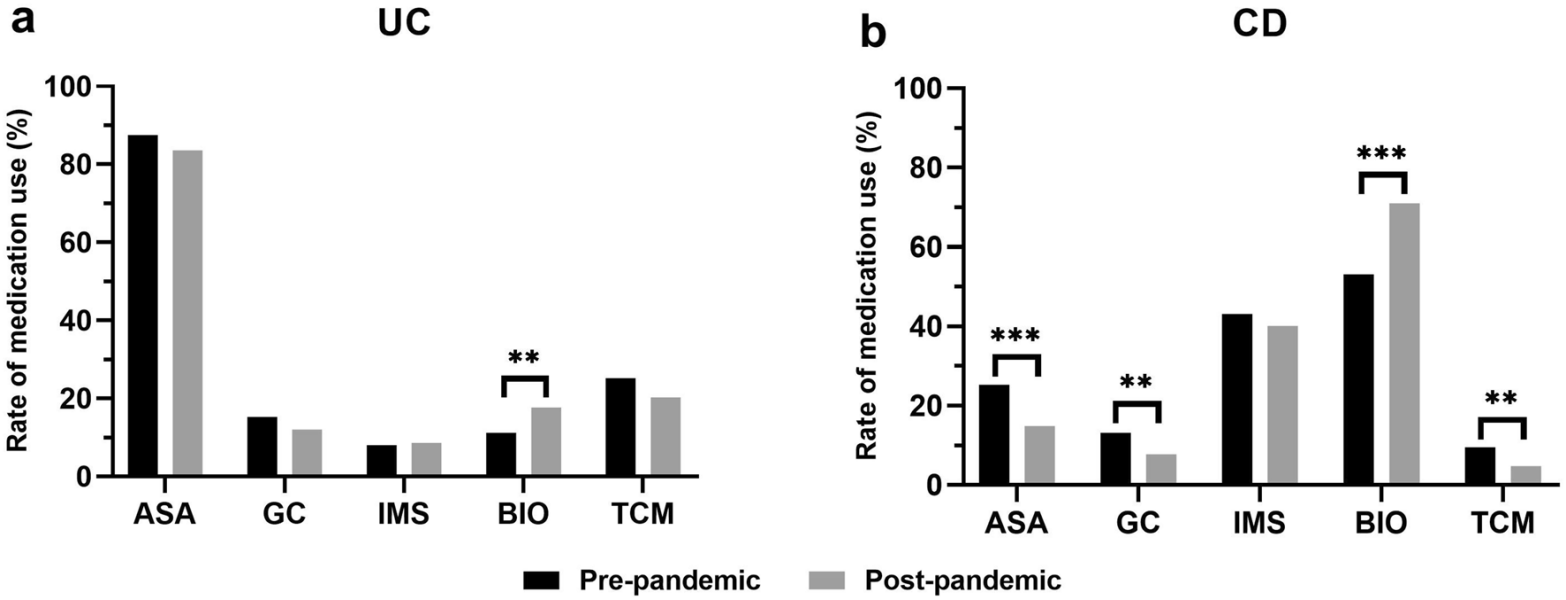
The change of drugs used before and after outbreak. (**a**) The change of drugs used in UC patients; (**b**) The change of drugs used in CD patients. ASA: Aminosalicylic acid; GC: glucocorticoid; IMS: immunosuppressants; BIO: biologics; TCM: Traditional Chinese medicine.

Figure 3 presented the use of therapeutic agents for UC and CD before and after the outbreak. Aminosalicylic acid(ASA) were the most frequently used drugs in the treatment of UC, with a high use rate of 85.7% and 83.6% before and after the outbreak, respectively (*P* = 0.362). Traditional Chinese medicine (TCM) was the second most commonly used drug, reaching 25.7% before the outbreak, although this difference was statistically insignificant (*P* = 0.051) when it decreased to 20.3% after the outbreak. The proportion of glucocorticoid (GC) decreased from 15.3 to 12.0% (*P* = 0.300), while the proportion of immunosuppressants increased slightly from 8.0 to 8.6% (*P* = 0.721). There was a significant increase in the use of biologics, from 11.2% to 17.7% (*p* = 0.005). For CD, biologics were the most frequently used therapeutic agents, with a significant increase from 53.2 before the outbreak to 71.0% after the outbreak (*P* < 0.001). Immunosuppressants were the second most commonly used drug, with a decrease in the proportion from 43.1 to 40.1%, with no statistical difference (*P* = 0.322). There was a significant decrease in the proportion of ASA (25.3% vs. 14.9%, *P* < 0.001) and GC (13.2% vs. 7.8%, *P* = 0.004). Compared to the high percentage in UC, the proportion of TCM in the treatment of CD was not high, at 9.5% before the outbreak and decreasing to 4.8% after the outbreak (*P* = 0.003).

**Figure 4:**
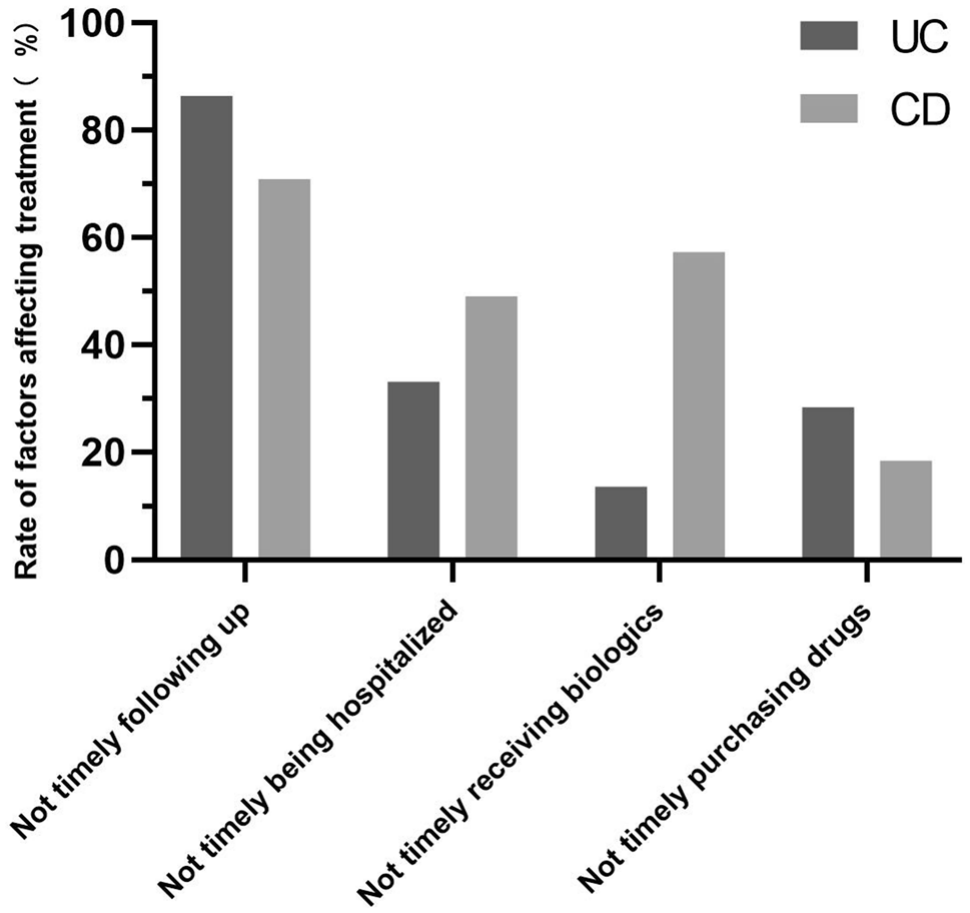
Impact of the pandemic on the treatment of patients with IBD.

Three hundred and seventy-five (37.5%) patients (UC: 169, CD: 206) considered that the pandemic had an impact on their treatment. As shown in Fig. 4, the biggest problem was unable to follow up timely. In CD, not timely biologic treatment and not timely hospitalization were two other important aspects, with 49.0% and 57.3% respectively. In UC, not timely hospitalization and not timely purchasing drugs were two other important aspects, and the rate were at 33.1% and 28.4% respectively. Three hundred and thirty-two patients (33.2%) had experienced an exacerbation of their disease after the outbreak.

In a logistic regression analysis of factors that may have contributed to the impact of the pandemic on treatment, we included factors such as age, sex, diagnosis, duration of disease, and drug used before the pandemic. In univariate and multifactorial analyses, the use of GC before the pandemic was the only risk factor for the impact of the epidemic on treatment (OR = 1.446, *P* = 0.044).

**Figure 5:**
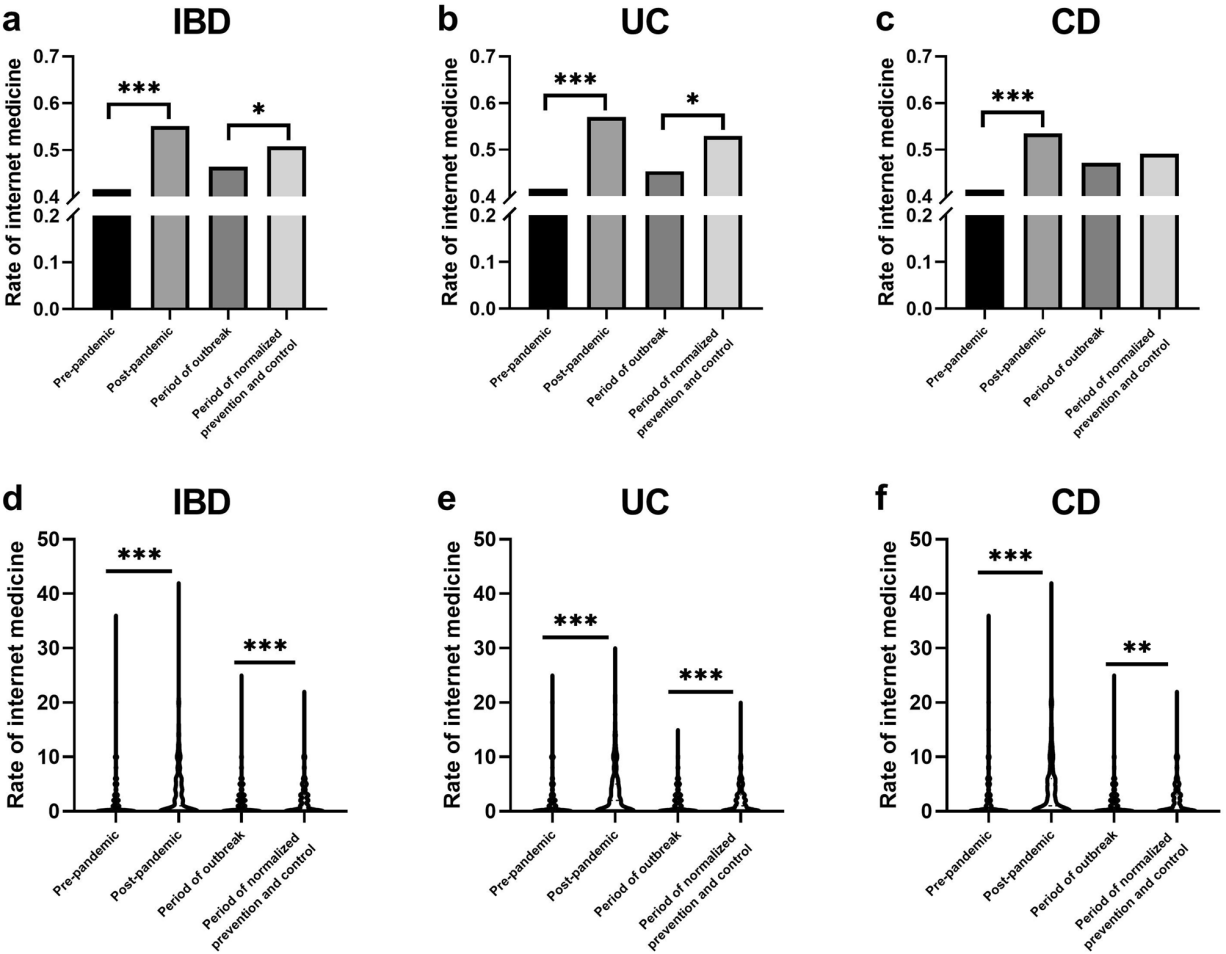
Internet medical service in IBD patients. (**a**) the rate of internet medical service in IBD; (**b**) the rate of internet medical service in UC; (**c**) the rate of internet medical service in CD; (**d**) the frequency of online medical service in IBD; (**e**) the frequency of online medical service in UC; f: the frequency of online medical service in CD.

In terms of internet medical service (Fig. 5), 41.6% of patients used the online medical service before the outbreak, and this increased to 55.1% after the outbreak (*P* < 0.001). We also observed a lower proportion during the outbreak than in the stage of the normalized prevention and control (50.8% vs. 46.4%, *P* = 0.044) and similarly in UC patients (52.9% vs. 45.4%, *P* = 0.021), however, the elevated proportion was not significant in CD (49.1% vs. 47.2%, *P* = 0.542). Significant increases in the frequency of online medical service were observed at each period of time. In a subgroup analysis of people with and without online medical service, there were no differences in basic characteristics such as age, education level, diagnosis, and disease duration, except for a significantly higher proportion of women than men who used online medical service (63.9% vs. 56.6%, *P* = 0.020). Although e-visits are gradually being used more and more, our survey found that 82.8%(829/1001) patients still preferred face-to-face visits.

## Discussion

The COVID-19 pandemic had profoundly affected people’s lives worldwide for more than 3 years. The response to the pandemic varies from country to country, because of differences in culture and values. China had adopted the rigorous dynamic zero-COVID strategy. However, recurrent outbreaks and the regular pandemic prevention and control policy, especially travel restrictions, had an impact on the long-term treatment and follow-up of IBD patients. So our study firstly explored the health care service of IBD patients after the first outbreak under the dynamic zero-COVID strategy.

Studies from early outbreaks in China showed that 23%–27.7% of patients experienced medication adjustment or discontinuation^3,4^. Our study showed that 67% of patients felt stable after the outbreak, and about 75% of patients did not have an adjustment in their treatment, which is consistent with the results of the previous studies. Whether at the beginning of the outbreak or the stage of implementing the normalized pandemic prevention and control policy, treatment options for most patients had not been affected. ASA, immunosuppressants agents and biologics are all recommended for continued treatment of IBD without SARS-CoV-2 infection, with the exception of glucocorticoids, according to the consensuses on the management of IBD patients during the pandemic published by various professional organizations^5–7^. Therefore, we observed that there was a significant increase in the use of biologics after the outbreak of COVID-19. The reduced proportion of GC in our study was consistent with widely accepted perception of reducing the dose or discontinuing the use of glucocorticoid to prevent SARS-CoV-2 infection. At the same time, in our analysis, patients using GC were more likely to be affected by the pandemic. Considering the side effects of long-term, the use of GC in the treatment of IBD should be more cautious, and other therapeutic drugs could be administered according to the principles of treatment.

In addition, 37.5% of the patients in our study were affected in not being able to make timely follow-up appointments, not being able to be hospitalized on time, not receiving biologics on time and not being able to purchase the medications they needed. Previous studies had shown that outpatient visits for IBD were significantly reduced during the pandemic^4,8,9^, because social distancing and lockdown had been central to reducing the transmission rate of SARS-CoV-2. On one side, healthcare service providers reduced the number of outpatient visits, and on the other side, travel restrictions made it hard to see doctors, meanwhile colonoscopy, an important screening test, was not offered except in emergencies, all of which made the inability to follow up and access to precision medicine timely the biggest concern for IBD patients during the pandemic. The recurrence of the pandemic also disrupted patients’ previous regular treatment schedule, so we observed an increase in the number of outpatient visits per capita after the outbreak. Meanwhile, there was a significant increase in hospitalization rate for CD, which was consistent with the result of the remarkable increase in the use of biologics. As a result, there has been a significant increase in the medical burden on CD patients. However, although UC patients showed an increase in the frequency of visits and hospitalizations, the impact of this change on the disease burden was not as pronounced as in CD patients. This difference between the two populations was related to the current differences in the drug treatment of UC and CD in China. ASA remains the predominant therapeutic agent in the treatment of UC, and an ASA-centered optimized treatment regimen could help most UC patients achieve improvement or remission of clinical symptoms. In contrast, given the complexity and intractability of CD, biologics have become the predominant drug for CD. In China, infliximab (IFX, an intravenous anti-tumor necrosis factor-α agent) is the only drug which can be reimbursed by medical insurance in IBD biologic treatment until 2021. So IBD patients treated with biologics other than IFX would still have bigger financial burden. This would explain the increased burden of disease was more obvious in CD patients in our study. However, ustekinumab, adalimumab and vedolizumab had been approved in 2022 in China to be reimbursed by medical insurance, which meant that the burden of people with IBD would be greatly alleviated.

Although the pandemic had a dramatic impact on the operation of the healthcare system, only less than 20% of patients had their hospitalization delayed for more than 1 week. This proportion did not show a large change, either before or after the pandemic. This had been closely linked to the development of day-care ward. With the accumulation of experience in the use of biological agents, Chinese IBD doctors simplified the treatment process of IBD, and used the day-care ward which were an efficient and convenient way to ensure the treatment of IBD patients. Therefore, in our study, we did not observe a significant delay in hospitalization for IBD after the outbreak. Although the day-care ward model was a good model for maintaining therapy, it was only implemented in some large centers. Therefore delaying of treatment during outbreaks was also of concern. As more easy-to-use biologics which are administered subcutaneously or orally are being applied in the treatment of IBD, the continuity of biologic therapy for IBD patients might be more assured, especially during a special time of major public health events such as COVID-19.

After the outbreak of the COVID-19, British Society of Gastroenterology (BSG) firstly distributed a position statement on SARS-CoV-2 vaccination for patients with inflammatory bowel disease, recommending vaccinating against SARS-CoV-2 for IBD patients^10^. Soon afterwards, an international consensus from the International Organization for the Study of Inflammatory Bowel Diseases (IOIBD) supported this viewpoint^11^. Previous study had shown that SARS-CoV-2 vaccine was safe for IBD patients, with only 2.1% experiencing flaring of symptoms^12^. Considering both the protection offered by the vaccine and the risk of secondary or more infections, 95% of the physicians recommended SARS-CoV-2 vaccine for IBD patients and only 43% of patients were willing to receive the vaccine^13^. In China, however, the rate was even lower, at 35.5%. Chinese IBD patients were more concerned about flaring of the disease after vaccination. This big difference in perception between doctors and patients means that gastroenterologists need to put more effort into communicating with their patients, to eliminate this difference, and promote the implementation of public health strategies to safeguard public health.

Studies in Denmark and the Netherlands have demonstrated the safety of telemedicine combined with the ability to reduce the number of patient visits and hospitalizations and improve patient adherence^14–17^. During the pandemic, telemedicine may be an excellent alternative for IBD patients when face-to-face visits are not possible. A global questionnaire from the International Organization for the Study of Inflammatory Bowel Diseases showed an increase in telemedicine during the pandemic, when face-to-face visits were significantly reduced^18^. Given the uneven distribution of medical resources and the pandemic travel restrictions in China, telemedicine is an ideal way to provide quality medical service to IBD patients. Previous studies in China had showed that during the pandemic, the proportion of gastroenterologists who use telemedicine increased from 46.13% before the pandemic to 64.98%, with WeChat (64.23%), third-party online clinics (59.12%), and hospital online clinics (27.74%) being the most commonly used online platforms^9^. Our study also found the similar increase of online medical service after the outbreak. This result indicated a gradual increase in the demand for telemedicine after the pandemic. Unfortunately, the preferred mode of medical visit for 82.8% of IBD patients in our study was still the face-to-face visit, consistent with previous study that 90.24% of gastroenterologists and 75.25% of IBD patients preferred face-to-face visit^9^. We think this result is partly due to the limitations of telemedicine itself. Firstly, gastroenterologists are unable to get sufficient interaction with patients from the online platform, and thus get incomplete information. Secondly, Chinese doctors have a large workload and do not have enough time to devote to online medicine to play its role. Finally Chinese patients are used to face-to-face visits and do not completely trust telemedicine, as well as the costs of online medical service are not covered by health insurance. Complexity of operation is also a barrier to using telemedicine. As a result, telemedicine has not gained sufficient acceptance by physicians and patients, and is only a substitute when face-to-face visits are not possible. Therefore, the construction of a more standardized and unified telemedicine platform easily to operate is necessary, and there is also a need to encourage more physicians and patients to participate in the practice of telemedicine. Telemedicine will play an important role in the healthcare system in future and be an effective initiative that can minimize the impact of major public health events on the management of IBD patients.

Our study also has some limitations. Firstly, this was a cross-sectional study, and recall bias and quality control of the questionnaire were inevitable problems. Due to the lack of a nationwide platform for the management of IBD patients in China, it was difficult to aggregate some exact clinical data from different centers. Pre-survey of a small sample was conducted before the formal start of the study, and the questionnaire was modified based on the feedback to facilitate patient to understand and complete the questionnaire to minimize the associated bias. Secondly, we only included patients diagnosed before 2019, and patients who were newly diagnosed after the outbreak were not considered, so that the impact of the pandemic on them might require additional studies to explore. Thirdly, our study was carried out online, which may not be exempt from selection bias given that the enrolled participants were mostly those with internet access. With the development of information technology, smartphones play an important role in eating, traveling, shopping, etc. It can be said that smartphones have been popularized among the general public. Meanwhile, inflammatory bowel disease patients are highly prevalent in young adults, most of whom are proficient in using the Internet and smartphones. This selection bias was indeed a possible influence, but based on the above practical considerations, we thought that the impact of this bias was relatively small. In the end, the severity of the pandemic and the prevention and control measures vary from country to country, even from city to city, and this study can only show the changes in healthcare services for Chinese IBD patients after the outbreak, whether the findings can be generalized to other countries is uncertain.

## Conclusion

China’s dynamic zero-COVID strategy and strict measures had a long-term impact on medical care service for IBD patients. Our study showed that the treatment of approximately one-third of patients was significantly affected by the pandemic, with IBD patients experiencing varying increases in the frequency of outpatient visits, hospitalizations, and hospitalization rates, and CD patients also facing an increased financial burden of disease. Biologics had become the treatment for more patients, especially for CD. Meanwhile the demand for telemedicine had increased significantly, but face-to-face visits were still the first preferred healthcare mode for IBD patients. How to make full use of network technology, improving and popularizing telemedicine, and helping patients acquire better health care services during major public health events, is still an important question that we need to think deeply about.

### Supplementary Information

Supplementary Information.

## Data Availability

The datasets used or analyzed during the current study are available from the corresponding author on reasonable request.
