# Supplementary material for: The change of healthcare service in Chinese patients with inflammatory bowel disease during the pandemic: a national multicenter cross-sectional study
Source: Sci Rep. 2023 Nov 16;13:20084. doi: 10.1038/s41598-023-46892-5 (PMC10654896; doi:10.1038/s41598-023-46892-5)
Supplement: Supplementary file 1 — Supplementary Information. [file 41598_2023_46892_MOESM1_ESM.docx]

**Questionnaire on the impact of the COVID-19 pandemic on diagnosis and treatment in Chinese IBD patients during 2019-2020**

To understand the impact of the COVID-19 pandemic on the management of IBD patients in China, we conduct a study on the impact of the pandemic on the treatment of Chinese IBD patients through a questionnaire survey. The questionnaire consists of 43 items, with three main sections: general information, disease-related information, and treatment-related information. We cordially invite you to participate in this study. Participation in this study is at your own discretion and is entirely voluntary. If you decide not to take part in the study, you will not be penalised or lose any benefits you may have. You may also choose to withdraw from the study at any time. If you have decided to participate in this study, please sign this informed consent form to indicate your agreement and complete the questionnaire truthfully. The information you provide will be treated in absolute confidence. The study data will only be used for clinical research and will not be used for any commercial purposes. We will publish the results of the study in national or international medical journals. All publications will not disclose any of your personal data. If you have any questions, you can contact Dr. Liang Jie, (029-84771535), the leaders of this study at Xijing Hospital of Digestive Diseases. Thank you for your participation.

□ Yes, I **agree** to participate in this study

□ No, I **disagree** to participate in this study

**Part 1: General information**

1. Name: ________________

2. Age: _________________

3. Phone number: _______________________

4. Sex: □ male □ female

5. Marriage: □ single □ married

6. Educational level: □ high school and below □ college and above

7. Address: province city

**Part 2: Disease-related information**

1. Your diagnosis

□ Ulcerative colitis(UC)

□ Crohn’s disease(CD)

2. Duration of your IBD

□ 1-4 years

□ 5-9 years

□ 10-14 years

□ ≥15 years

3. The hospital you are currently attending is:

_________________________________

4. Have you been infected with SARS-CoV-2?

□ Yes

□ No

5. Has anyone in your family been infected with SARS-CoV-2?

□ Yes

□ No

6. Have you vaccinated or are you planning to vaccinate against COVID-19?

□ Yes

□ No

7. Which of the following issues concerns you more?

□ Poor control of IBD

□ Infection with SARS-CoV-2

8. Are you worried about your family knowing you have IBD?

□ Yes, worried that I would become a burden to them

□ No, not worried about this

9. Are you worried about your friends and colleagues knowing that you have IBD?

□ Yes.

□ No.

10. Are you worried that your employer will know that you have IBD and that it would affect your work?

□ Yes.

□ No.

11. Do you have confidence in life despite your IBD?

□ Yes.

□ No.

12. Have you experienced an exacerbation of your symptoms after the pandemic outbreak?

□ Yes, suffering from the exacerbating disease.

□ No, maintaning a stable disease.

13. Whether the treatment of IBD was affected by the pandemic outbreak?

□ Yes. (If yes, then go to quession 14)

□ No. (If no, then go to quession 15)

14. Your treatment of IBD has been affected by the pandemic, specifically because of: (**multiple answers possible**)

□ Not timely following up.

□ Not timely hospitalization.

□ Not timely biologic treatment.

□ Not timely purchasing therapeutic drugs.

15. Which model of therapy would you prefer for the treatment of IBD?

□ Western medical treatment

□ Traditional Chinese Medicine treatment

□ Integrated Chinese and Western Medicine treatment

16. Which of the following is more of a concern to you in the treatment of IBD?

□ Poorly controlled disease

□ Expensive treatment cost

□ Drug side effects

**Part 3: Treatment-related information**

1. Your therapeutic regimens for IBD in **2019** included: **(multiple answers possible)**

□ **Aminosalicylic acid**: mesalazine, olsalazine, sulfasalazine, etc.

□ **Glucocorticoid**: prednisone, hydrocortisone, methylprednisone, etc.

□ **Immunosuppressant**: azathioprine, methotrexate, thalidomide, cyclosporine, etc.

□ **Biologic**: infliximab, adalimumab, vedolizumab, ustekinumab, etc.

□ **Tranditional Chinese medicine**

□ **Surgery**

2. Your therapeutic regimens for IBD in **2020** included: **(multiple answers possible)**

□ **Aminosalicylic acid**: mesalazine, olsalazine, sulfasalazine, etc.

□ **Glucocorticoid**: prednisone, hydrocortisone, methylprednisone, etc.

□ **Immunosuppressant**: azathioprine, methotrexate, thalidomide, cyclosporine, etc.

□ **Biologic**: infliximab, adalimumab, vedolizumab, ustekinumab, etc.

□ **Tranditional Chinese medicine**

□ **Surgery**

3. Your annual cost for IBD treatment in **2019** was approximately:

□ ≤10 thousands Yuan/year

□ ≤30 thousands Yuan/year

□ ≤50 thousands Yuan/year

□ ≤80 thousands Yuan/year

□ ＞80 thousands Yuan/year

4. Your annual cost for IBD treatment in **2020** was approximately:

□ ≤10 thousands Yuan/year

□ ≤30 thousands Yuan/year

□ ≤50 thousands Yuan/year

□ ≤80 thousands Yuan/year

□ ＞80 thousands Yuan/year

5. The number of **outpatient visits** you made in **2019** for IBD was:

times

6. The number of **outpatient visits** you made in **the first half of 2020** for IBD was:

times

7. The number of **outpatient visits** you made in **the second half of 2020** for IBD was:

times

8. The number of **hospitalization** you had in **2019** for IBD was:

times

9. The number of **hospitalization** you had in **the first half of 2020** for IBD was:

times

10. The number of **hospitalization** you had in **the second half of 2020** for IBD was:

times

11. The number of **surgery** you had in **2019** for IBD was:

times

12. The number of **surgery** you had in **the first half of 2020** for IBD was:

times

13. The number of **surgery** you had in **the second half of 2020** for IBD was:

times

14. Your waiting time for hospitalisation for IBD in **2019** was:

□ On time

□ Delayed within 1 week

□ Delayed within 1 month

□ Delayed more than 1 month

15. Your waiting time for hospitalisation for IBD in **the first half of 2020** was:

□ On time

□ Delayed within 1 week

□ Delayed within 1 month

□ Delayed more than 1 month

16. Your waiting time for hospitalisation for IBD in **the second half of 2020** was:

□ On time

□ Delayed within 1 week

□ Delayed within 1 month

□ Delayed more than 1 month

17. The number of online consultations (WeChat, web consultations, online meetings, etc.) you have had with a gastroenterology specialist or IBD specialist for IBD in **2019** was:

times

18. The number of online consultations (WeChat, web consultations, online meetings, etc.) you have had with a gastroenterology specialist or IBD specialist for IBD in **the first half of 2020** was:

times

19. The number of online consultations (WeChat, web consultations, online meetings, etc.) you have had with a gastroenterology specialist or IBD specialist for IBD in **the second half of 2020** was:

times

20. Your preferred method of visit is

□ Face to face visit

□ Online visit
